# Anterior cruciate ligament reconstruction with quadriceps tendon-patellar bone allograft: matched case control study

**DOI:** 10.1186/s12891-018-1959-0

**Published:** 2018-02-09

**Authors:** Yoon-Ho Kwak, Sahnghoon Lee, Myung Chul Lee, Hyuk-Soo Han

**Affiliations:** Seoul National University Hospital, Seoul, Republic of Korea

**Keywords:** Arthroscopy, Anterior cruciate ligament, Quadriceps tendon-patellar bone, Allograft, Autograft

## Abstract

**Background:**

Quadriceps tendon-patellar bone (QTPB) autograft is an excellent graft option with good clinical outcome. Use of QTPB autografts have increased because they minimize donor-site morbidity including anterior knee pain, while providing adequate mechanical strength. Although, there were many clinical results about allografts that used in anterior cruciate ligament (ACL) reconstruction, it have never been reported about the clinical outcome of ACL reconstruction with QTPB allograft.

The purpose of this study is to evaluate the clinical outcome of ACL reconstruction with QTPB allograft and to compare with QTPB autograft. We hypothesized that ACL reconstruction with QTPB allograft had good functional outcomes and stability and no significant difference compared to the ACL reconstruction with QTPB autograft.

**Methods:**

From February 2009 to January 2014, 213 cases who received ACL reconstruction with QTPB grafts were included. Forty-five patients who received ACL reconstruction with QTPB allograft were individually matched in age, sex, direction of the injured knee and body mass index (BMI) to a control group of 45 patients who received QTPB autograft. Clinical results were evaluated using International Knee Documentation Committee (IKDC) score, Lysholm score, Tegner scale, Knee injury and Osteoarthritis Outcome Score (KOOS) and ligament laxity. An average follow-up time was 31.2 months.

**Results:**

The functional scores and ligament laxity improved from initial to the last visit in those with ACL reconstruction with QTPB allograft (*p* < 0.05). No significant statistical difference was found in clinical outcomes and complications including re-rupture between the QTPB allograft and autograft groups (*p* > 0.05). Laxity using anterior drawer test, Lachman test and KT-2000 showed no significant difference. No significant difference was found between the two groups in quadriceps peak extension torque, except at 60° per second at 6 months.

**Conclusion:**

QTPB allograft achieved good clinical outcome with no difference compared with QTPB autograft. QTPB allograft for ACL reconstruction is promising alternative to selected and compliant patients. Long-term follow-up needs to further evaluate the clinical outcomes and complications including re-rupture rate.

## Background

ACL reconstruction can be performed using several kinds of autograft or allograft tissue. Although, some recent research showed ACL reconstruction with autograft leads to lower retear rates in younger individuals [1], whether the outcomes of these two graft materials differ significantly is unclear [2–4] and the choice of the optimal graft for ACL reconstruction remains still controversial.

Good clinical results of ACL reconstruction have been achieved using proper graft materials, such as bone-patella tendon-bone (BPTB) or hamstring tendons, as well as quadriceps tendon-patellar bone (QTPB) [5–9]. The QTPB autograft is long established as a viable graft option with good clinical outcome [7, 10–18]. The use of QTPB autografts has increased in recent years because they minimize donor-site morbidity including anterior knee pain, while providing adequate mechanical strength as a graft [7, 12, 19, 20]. Several reports have suggested a biomechanical test for quadriceps tendon is comparable to that for BPTB [21–23]. However, QTPB allograft has been the least studied. Previous studies have compared other allografts with autografts in primary ACL reconstruction with results showing inconsistent clinical equivalency [16, 24, 25] and no study has directly compared QTPB allograft to autograft.

The purpose of this study is to evaluate the clinical outcomes of ACL reconstruction with QTPB allograft regarding anteroposterior knee stability, activity, and functional scores. We also evaluated whether the outcomes differed with QTPB allograft and autograft used for ACL reconstruction. We hypothesized that ACL reconstruction with QTPB allograft had good functional outcomes and stability and no significant difference compared to the ACL reconstruction with QTPB autograft.

## Methods

**Fig. 1:**
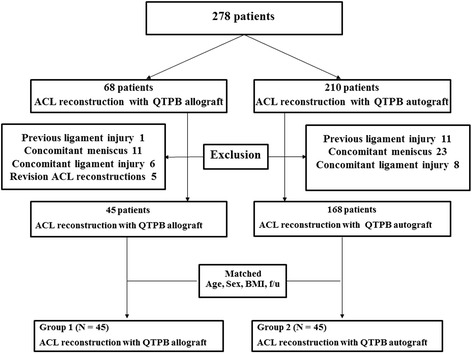
Flow diagram of patients screened and grouped

This is a retrospective study with ethically approved by the institutional review board of Seoul National University Hospital (No. H-1604-033-753)**.** From February 2009 to January 2014, 278 patients diagnosed as ACL total ruptures who received ACL reconstruction with QTPB grafts were screened. The choice of the graft was determined by full discussion between the patient and the physician. We included patients followed-up more than 2 years after ACL reconstruction. Exclusion criteria were patients who had previous ligament injury and who had concomitant meniscus or ligament injury of the affected knee, except for a Grade I or II medial collateral ligament injury. Revision ACL reconstructions were also excluded. Finally, 45 patients who had QTPB allografts and 168 patients who had QTPB autografts met these criteria. The 45 patients in the QTPB allograft group were matched for age and body mass index (BMI) with 45 patients in the QTPB autograft group (Fig. 1).

Ligament laxity was evaluated with anterior drawer test, Lachman test, pivot shift test and a KT-2000 arthrometer (MedMetric Inc., San Diego, CA) preoperatively, postoperatively at 1, 3 and 6 months and annually thereafter. Quadriceps peak extension torque was checked at 60° and 180° per second using an isokinetic testing device (Cybex, Ronkonkoma, NY) at 6, 12 and 24 months. Functional outcomes including International Knee Documentation Committee (IKDC) score [26], Lysholm Knee Score [27], Tegner score [28] and Knee Injury and Osteoarthritis Outcome Score (KOOS) [29] were evaluated preoperatively and at the postoperative follow-ups.

**Fig. 2:**
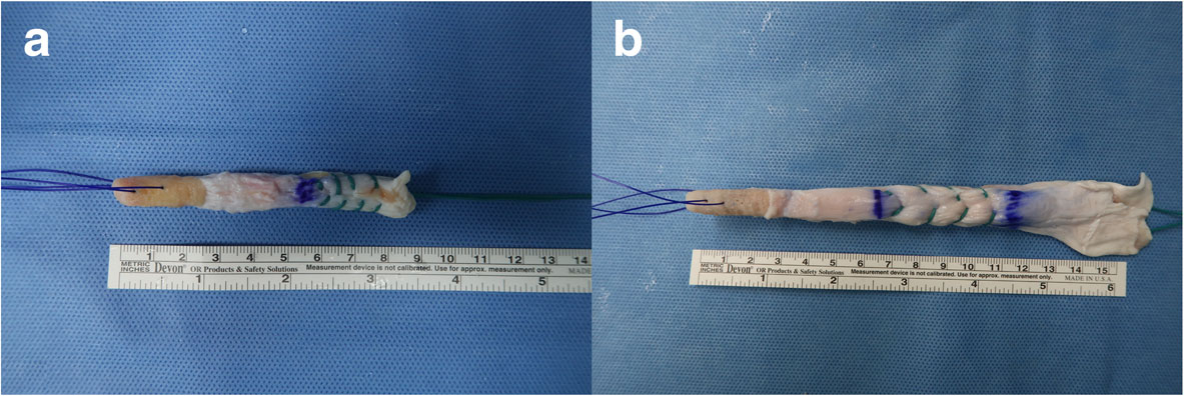
Quadriceps tendon-patellar bone autograft (**a**) and allograft (**b**)

QTPB allografts were provided by Community Tissue Services (Kettering, OH), a certified soft tissue bank. Allografts were the non-gamma irradiated fresh frozen type. Serological and microbiological tests were performed on the donors in accordance with American Association of Tissue Bank (AATB) standards. On the day of surgery, the allograft was transported from the local distributor to the operating room adding dry ice for below zero temperature conditions (− 70 to − 60 °C). The state of packaging and expiry dates were checked before use and the grafts soaked in sterile saline, warmed to 37 °C for 30 min. A trapezoidal bone block measuring 10 mm in width, 20- to 25 mm in length and 7 mm in thickness was obtained using an oscillating saw. A strip of the quadriceps tendon measuring 10 mm in width, 6-8 mm in thickness and 6 cm in length was excised from the proximal portion of the patellar bone block (Fig. 2).

The QTPB autograft was harvested through a 4 cm midline incision centered over the patella proximal border and prepared by the same method of used for the QTPB allograft. We were cautioned not to approach the suprapatellar pouch by saving part of the vastus intermedius tendon. If the suprapatellar pouch was damaged, the synovial lining was repaired with an absorbable suture. Superficial layers of the cut surface of the tendon were closed transversely with absorbable sutures and the defect was left as a potential space. The bone defect was left in empty space. A hole was drilled in the bone block from the patella base and two absorbable sutures were passed through. The tendinous portion of the graft was secured with two Number 5 Ethibond™ sutures (Ethicon Inc., Somerville, NJ) using the Krackow method with an extension of approximately 30 mm (Fig. 2).

After a graft had been prepared, ACL reconstruction was performed by the modified transtibial technique [30]. A tibial tunnel 10 mm in diameter was drilled and the intra-articular opening of the tunnel was placed in the center of the ACL attachment using an ACL endoscopic guide system (Smith and Nephew, Inc., Andover, MA). A femoral tunnel that was also 10 mm in diameter was drilled through the tibial tunnel in the 10:30 to 11 o’clock position for the right knee. The posterior cortex of the femoral tunnel was approximately 2 mm thick. Notchplasty was performed to prevent graft impingement if needed. After the graft had been passed through the femoral tunnels, a 8 mm diameter**,** 25 mm length metal interference screw (Linvatec, Largo, FL) was used to fix the bone block on the femoral side. The ACL reconstructed knee was moved in flexion and extension 15 to 20 times through a full range of motion under tensioning the graft. The tendinous portion was fixed on the tibial side with a 10 mm diameter, 25 mm length metal interference screw (Synthes, West Chester, Pennsylvania) augmented by tying sutures over a cortical screw with the knee extended.

The same rehabilitation protocol was applied for both groups. Patients were taught quadriceps setting exercise and straight leg raising prior to surgery and exercise commenced soon after surgery. Kinetic exercise and weight-bearing progressed as tolerated. Passive range of motion of the ACL reconstructed knee was started from 45° knee flexion and full extension within 3 days after surgery. Patients put on the ACL knee brace 1 week after surgery when swelling decreased. An ACL brace set at 0° to 90° was worn for 3 weeks and then set at 0° to full flexion for an additional 3 weeks postoperatively. Full flexion was allowed at postoperative 7 weeks. Patients usually returned to normal daily activity 3 months after ACL reconstruction and strenuous exercise was approved 6 months postoperatively.

We used SPSS for Windows version 20.0 (SPSS Inc., Chicago, IL) for statistical analyses. The independent t-test was used for the comparison of continuous variables (IKDC score, Lysholm score, Tegner score, KOOS score, extensor strengths and KT-2000 arthrometry), and the chi-squared test was used for the categorical variables (grades of ligament stability including anterior drawer test, Lachman test, pivot shift test). Paired t-test was used for comparing the data before and after the ACL reconstruction. The significance level was set at *P* < 0.05. A post-hoc analysis was performed by G-Power, and confirmed 42 patients in each group to detect one standard deviation difference at 80% power. The ligament laxity checked by KT-2000 was primary outcome in which the sample size was based. This study was approved by the institutional review board.

## Results

As we mentioned above, 45 patients in each groups were included in this retrospective study. An average follow-up time was 31.2 months.

There were no differences in preoperative demographic data between the two groups (Table 1). Comparisons of knee laxity and clinical outcome between two groups are summarized in Tables 2 and 3. According to the anterior drawer test, Lachman test, and pivot-shift test, there was no significant difference between the two groups preoperatively and at final follow-up (Table 2). All grades of instability were improved from the initial to final visit in both groups (*P* < 0.001). The mean side-to-side differences in anterior laxity during manual maximum testing using KT-2000 arthrometry were similar in the QTPB allograft and autograft groups preoperatively (4.8 ± 1.9 and 4.5 ± 1.8 mm; *P* = 0.370) and postoperatively (1.8 ± 1.6 mm and 1.4 ± 1.2 mm; *P* = 0.458). The KT-2000 measurements at postoperative 2 years follow-up were significantly improved than at preoperative in both groups (both *P* < 0.001).

**Table 1:** Patient demographic data

	Allograft group (*n* = 45)	Autograft group (*n* = 45)	*p-value*
Age^a^	34.5 ± 12.8	34.5 ± 12.8	1.000
Sex (Male/Female)	38/7	38/7	1.000
Right/Left	20/25	22/23	0.833
BMI (kg/m^2^)^a^	25.2 ± 4.0	25.3 ± 4.5	0.905
F/U (months)**	32.6 ± 7.4 (27.5 – 39.5)	29.8 ± 6.5 (24.9 – 44.3)	0.300

Values are expressed as mean ± standard deviation^a^ or mean ± standard deviation (range)**

**Table 2:** Evaluation of knee instability

	Preoperative			Postoperative 2 years		
	Allograft group	Autograft group	*p-value*	Allograft group	Autograft group	*p-value*
Anterior drawer test			0.826			0.652
Grade 0	5 (11.1%)	4 (8.9%)		29 (64.4%)	32 (71.1%)	
Grade 1	16 (35.6%)	15 (33.3%)		16 (35.6%)	13 (28.9%)	
Grade 2	17 (37.8%)	21 (46.7%)		0 (0.0%)	0 (0.0%)	
Grade 3	7 (15.6%)	5 (11.1%)		0 (0.0%)	0 (0.0%)	
Lachman test			0.717			0.404
Grade 0	1 (2.2%)	3 (6.7%)		26 (57.8%)	29 (64.4%)	
Grade 1	17 (37.8%)	14 (31.1%)		18 (40.0%)	15 (33.3%)	
Grade 2	19 (42.2%)	19 (42.2%)		1 (2.2%)	1 (2.2%)	
Grade 3	8 (17.8%)	9 (20.0%)		0 (0.0%)	0 (0.0%)	
Pivot shift test			0.258			0.823
Grade 0	6 (13.3%)	4 (8.9%)		31 (68.9%)	29 (64.4%)	
Grade 1	16 (35.6%)	22 (48.9%)		14 (31.1%)	16 (35.6%)	
Grade 2	21 (46.7%)	14 (31.1%)		0 (0.0%)	0 (0.0%)	
Grade 3	2 (4.4%)	5 (11.1%)		0 (0.0%)	0 (0.0%)	
KT-2000 (mm)^a^	4.8 ± 1.9	4.5 ± 1.8	0.392	1.8 ± 1.6	1.4 ± 1.2	0.235
KT-2000 (No. of patients)						
< 3 mm	2 (4.4%)	5 (11.1%)		39 (86.7%)	34 (75.6%)	
3 – 5 mm	26 (57.8%)	30 (66.7%)		6 (13.3%)	11 (24.4%)	
> 5 mm	17 (37.8%)	10 (22.2%)		0 (0.0%)	0 (0.0%)	

^a^Values are expressed as mean ± standard deviation

**Table 3:** Outcomes of functional score

	Preoperative			Postoperative		
	Allograft group	Autograft group	*p-value*	Allograft group	Autograft group	*p-value*
IKDC score	42.3 ± 16.1	42.7 ± 22.6	0.928	70.1 ± 12.5	67.3 ± 16.8	0.366
Lysholm score	65.0 ± 9.1	62.4 ± 8.4	0.166	88.7 ± 6.4	87.0 ± 5.3	0.170
Tegner scale	3.2 [2-4.8]	2.8 [1.8-4]	0.203	7 [6.0-8.0]	7.2 [6.3-8.2]	0.434
KOOS	245.1 ± 87.5	273.8 ± 95.1	0.163	413.2 ± 40.6	423.1 ± 50.9	0.334

Values are expressed as mean ± standard deviation in IKDC score, Lysholm score, KOOS; Values are expressed as the median and interquartile ranges in Tegner scale

*IKDC* International Knee Documentation Committee, *KOOS* Knee injury and Osteoarthritis Outcome Score

Forty-one patients, and greater than 5 mm in 12 patients. One patient per group showed grade II in Lachman test, which generally considered clinical failure [31, 32]. However, anterior drawer test, pivot-shift test and KT-2000 measurements showed no instability and had no subjective instability in both 2 patients. Therefore, we decided not to have revision surgery.

No significant differences in functional scores including IKDC score, Lysholm score, Tegner score, KOOS were found between the two groups at preoperative and postoperative 2 years (Table 3). Mean preoperative functional scores in QTPB allografts group and autografts group were improved at postoperative 2 years follow-up (*p* < 0.001).

**Fig. 3:**
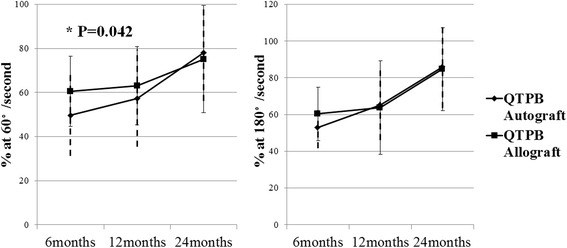
Side to side ratio of peak torque values by Cybex isokinetic testing at 60° (left) and 180° (right) per second. Vertical full line indicates the standard deviation of the peak extension torque in QTPB allograft group. Vertical dotted line indicates the standard deviation of the peak extension torque in QTPB autograft group. QTPB = quadriceps tendon patellar bone

Quadriceps peak extension torque at 60°and 180° per second increased with time at 6, 12, 24 months in both groups. No significant differences were found the two groups, except the value of the quadriceps peak extension torque at 60° per second at 6 months (*P* = 0.042) (Fig. 3).

In both groups, there were no postoperative complications during follow-ups such as arthrofibrosis, rerupture or infection. In the QTPB autograft group, two patients had paresthesia on the lateral side of the knee. The paresthesia completely disappeared about 2 months after ACL reconstruction. Two patients in the QTPB allograft group and three patients in the QTPB autograft group felt a clicking sensation in the knee during activities, and this symptom was relieved after an average of 3 months.

## Discussion

This is the first study comparing the knee stability and clinical outcomes of the QTPB allografts and autografts. The most important finding in this study was ACL reconstruction with QTPB allografts showed good clinical outcomes and had no significant differences compared with QTPB autografts. There was no difference about rerupture rate in short-term follow-up. However, 6 months after ACL reconstruction, quadriceps muscle power recovery was relatively good in ACL reconstruction with QTPB allograft.

Several studies have compared ACL reconstruction with QTPB autograft to other autografts and reported comparable results concerning knee stability and functional outcomes [10, 12–14, 19, 20]. Most clinical outcomes about ACL reconstruction with QTPB autograft in these studies were relatively good, which is also shown in our study**.**

Two studies have compared biomechanical properties of QTPB allograft to other grafts. One study compared the biomechanical properties of 12 QTPB allografts to 11 BPTB allografts [21]. The authors found that the cross-sectional area of the QTPB allografts was nearly twice that of the BPTB allografts and ultimate load to failure and stiffness was significantly higher for the QTPB allografts. The variability in the cross-sectional area was similar in both tendon groups. In the other study, quadriceps and Achilles tendon pairs from nine research-consented donors were tested [33]. All specimens were processed to reduce bioburden and terminally sterilized by gamma irradiation. The authors found that QTPB allografts displayed significantly higher displacement at maximum load and significantly lower stiffness than achilles allografts. Maximum stress, strain at maximum stress, modulus and cyclic elongation exhibited no significant differences between two tendon types. On the basis of these two biomechanical studies, QTPB allograft is judged to be a biomechanically qualified graft for ACL reconstruction.

Several studies have reported allograft rerupture rates were higher than autograft after ACL reconstruction. One study reported a 7% rate of late allograft traumatic rupture versus none in autografts [34]. Another study reported that allograft showed a threefold increase in rerupture rate relative to the autograft (12.7% vs. 4.3%) [35]. There are several possible explanations. Sterilization processes that influence remodeling of the allograft in vivo can cause a higher rate of rerupture in ACL reconstruction done with allograft ACL [36]. In addition, allograft patients may participate in a higher level of activity earlier after surgery, secondary to less pain including donor site pain, with more consequent stress on their grafts, than in autograft patients [37]. In this minimum 2-year follow-up study, there was no rerupture case in ACL reconstruction with QTPB allograft. However, long- term follow-up and further evaluation will be planned.

Although the QTPB autograft has less donor-site morbidity than other autografts, quadriceps graft harvest can cause temporal quadriceps weakness [14, 38–40]. In order to evaluate quadriceps muscle power, we used a Cybex isokinetic testing device. In our study, quadriceps peak extension torque at 60° per second in the QTPB autograft group at postoperative 6 months was less than in the QTPB allograft group. However, there was no significant difference in later follow-up.

In general, unlike primary reconstruction, in revision cases the choice of graft can be determined by the nature of the graft that was previously used, and an allograft may be an appealing situation to use [32]. ACL reconstruction with QTPB allograft showed good clinical results in this study, then also possible options in revision ACL reconstruction**.**

This study has some limitations. First, this study has a retrospective design and the patients were not assigned randomly, increasing selection bias. However, there were several strengths in this study, including the matched demographic features of these patients, same surgical techniques, fixation method and rehabilitation program, which increased the power of statistical results**.** Furthermore, this is the first study reporting the clinical outcome of QTPB allograft and matched case-control study compared with QTPB allograft. Second, our study includes a relatively small number of patients especially on allograft group and has a short-term follow-up period. According to one study [41], at least 100 patients were required to detect a difference for the majority of outcome measures, and over 800 to detect a difference in return to pre-injury activity level. Comparing to this study, our study has limitations. In order to overcome these limitations, long-term follow up, large scaled, randomized controlled study will be scheduled to confirm the efficacy of this study. Third, our study does not include MRI evaluation of reconstructed ACL to confirm the ligamentizations of ACL. However, we could make an assumption by clinical results including anterior drawer test, Lachman test, pivot shift test and a KT-2000 arthrometer.

## Conclusions

ACL reconstruction with QTPB allograft achieves good knee stability and functional outcomes with no difference compared with QTPB autograft at 2 years follow-up. Therefore, QTPB allograft for ACL reconstruction is promising alternative to selected and compliant patients. Long-term follow-up needs to further evaluate the clinical outcomes and complications including re-rupture rate.

## Abbreviations

AATB: American Association of Tissue Bank
ACL: Anterior cruciate ligament
BPTB: Bone-patella tendon-bone
IKDC: International Knee Documentation Committee
KOOS: Knee injury and Osteoarthritis Outcome Score
QTPB: Quadriceps tendon-patellar bone
